# Revisiting exercise-induced premature ventricular complexes as a prognostic factor for mortality in asymptomatic patients: A systematic review and meta-analysis

**DOI:** 10.3389/fcvm.2022.949694

**Published:** 2022-09-29

**Authors:** Mohammad Iqbal, Iwan Cahyo Santosa Putra, William Kamarullah, Raymond Pranata, Chaerul Achmad, Giky Karwiky, Miftah Pramudyo, Hanna Goenawan, Mohammad Rizki Akbar, Arief Sjamsulaksan Kartasasmita, Young Hoon Kim

**Affiliations:** ^1^Department of Cardiology and Vascular Medicine, Faculty of Medicine University of Padjadjaran, Bandung, Indonesia; ^2^Division of Cardiology, Department of Internal Medicine, Korea University Medical Center, Seoul, South Korea; ^3^R. Syamsudin, SH Regional Public Hospital, Sukabumi, West Java, Indonesia; ^4^Division of Physiology, Department of Biomedical Sciences, Faculty of Medicine University of Padjadjaran, Bandung, Indonesia; ^5^Faculty of Medicine University of Padjadjaran, Bandung, Indonesia

**Keywords:** electrocardiography, premature ventricular complexes, exercise test, EI-PVCs, arrhythmia, mortality

## Abstract

**Background:**

Recent investigations suggest that premature ventricular complexes (PVCs) during an exercise test are associated with an elevated risk of mortality in asymptomatic individuals. However, given the small number of studies included, the association between these two entities in the asymptomatic population remains obscure. Our aim was to evaluate this matter.

**Methods:**

A comprehensive literature search was conducted utilizing several online databases up to April 2022. The study comprised cohort studies examining the relationship between exercise-induced premature ventricular complexes (EI-PVCs) and all-cause mortality (ACM) as well as cardiovascular mortality (CVM) in asymptomatic populations. To provide diagnostic values across the statistically significant parameters, we additionally calculated sensitivity, specificity, and area under the curve (AUC).

**Results:**

A total of 13 studies consisting of 82,161 patients with a mean age of 49.3 years were included. EI-PVCs were linked to an increased risk of ACM (risk ratio (RR) = 1.30 (95% confidence interval (CI) = 1.18–1.42); *p* < 0.001; *I*^2^ = 59.6%, *p*-heterogeneity < 0.001) and CVM (RR = 1.67 (95% CI = 1.40–1.99); *p* < 0.001; *I*^2^ = 7.5%, *p*-heterogeneity = 0.373). Subgroup analysis based on the frequency of PVCs revealed that frequent PVCs were similarly related to a higher risk of ACM and CVM, but not infrequent PVCs. Moreover, diagnostic test accuracy meta-analysis showed that recovery phase EI-PVCs have a higher overall specificity than exercise phase EI-PVCs regarding our outcomes of interest.

**Conclusion:**

EI-PVCs are correlated with a higher risk of ACM and CVM. When compared to the exercise phase, the specificity of PVCs generated during the recovery period in predicting interest outcomes is higher. As a result, we propose that the exercise ECG be utilized on a regular basis in middle-aged asymptomatic individuals to measure the frequency of PVCs and stratify the risk of mortality.

**Systematic review registration:**

[https://www.crd.york.ac.uk/prospero/display_record.php?RecordID=328852], identifier [CRD42022328852].

## Introduction

According to the 2019 European Society of Cardiology (ESC) guideline, an exercise electrocardiogram (ECG) is a valuable tool for confirming the diagnosis of coronary artery disease (CAD) in suspected patients, but it is not indicated in the asymptomatic population without suspicion toward CAD (1). The interpretation of this test to detect CAD is commonly known to rely substantially on the development of ST-segment deviation (2). However, premature ventricular complexes (PVCs), another ECG sign, can also develop during exercise ECG (3–5). After years of examining the predictive significance of PVCs during exercise ECG in CAD patients, researchers have discovered that this ECG marker is substantially linked with all-cause mortality (ACM) and cardiovascular mortality (CVM) (6).

Interestingly, PVCs were also prevalent during exercise ECG in the asymptomatic group without a pre-existing CAD, with a frequency ranging from 3.8 to 27.5% (7, 8). Previously, exercise-induced premature ventricular complexes (EI-PVCs) in the asymptomatic population were thought to be a harmless condition that did not increase the risk of mortality. Nonetheless, three meta-analyses published in 2016 by Lee et al., Kim et al., and Peng et al. demonstrated that EI-PVCs in an asymptomatic population significantly increased the risk of mortality compared to those with normal exercise ECG (6, 9, 10). Furthermore, subgroup analysis in Kim et al. study revealed that recovery-induced PVCs were substantially correlated with a higher risk of ACM (10). However, due to the limited number of studies included in the subgroup analysis and the fact that this meta-analysis was performed in 2016, the association between recovery-induced PVCs with mortality in the asymptomatic population remains equivocal. Therefore, the present meta-analysis has two primary objectives. Firstly, to evaluate the prognostic value of exercise and recovery-induced PVCs in increasing the risk of ACM and CVM in the asymptomatic population. Secondly, compare these two parameters to determine which one is the better predictor of similar outcomes.

## Methods

### Protocol and registration

This meta-analysis was recorded in the PROSPERO (International Prospective Register of Systematic Reviews) database under registration number CRD42022328852 and followed the Preferred Reporting Items for Systematic Reviews and Meta-analyses (PRISMA) standards.

### Search strategy

This systematic review and meta-analysis coveted to investigate whether EI-PVCs throughout exercise stress testing prognosticate mortality in both ACM and CVM in asymptomatic individuals. In favor of this aim, two independent investigators searched MEDLINE (via PubMed), Europe PMC, and ScienceDirect databases from inception to April 2022 with the search terms (“ventricular premature complexes” [MeSH Terms]) AND (“all-cause mortality” OR “cardiovascular mortality”). We modified every search term to match the restrictions of each database, and the full-text articles were rigorously evaluated by both authors in case there was disagreement about whether research should be included based on its title or abstract.

### Eligibility criteria and outcomes of interest

This study included both prospective and retrospective observational studies providing the occurrence of EI-PVCs in asymptomatic adult participants (age ≥ 18 years) who underwent exercise stress ECG testing, in conjunction with our primary outcomes of interest on clinically validated definitions of mortality, both prompted by any causes (ACM) and cardiovascular (CVM) origin. The studies must report the incidence of our primary outcomes in a comparative demeanor between patients with EI-PVCs to those without these arrhythmias (either during exercise or recovery phase of an exercise stress test). We excluded review articles, editorials, commentaries, case reports/series, meta-analyses, conference abstracts, and studies in languages other than English. The adjudicator would be the third investigator, and any discrepancies would be resolved through discussion.

### Study selection and data collection

The preliminary search and duplication elimination were carried out by two separate investigators. Titles and abstracts were reviewed for eligibility, and the full text of potentially eligible papers was evaluated using predetermined inclusion and exclusion criteria. Data extraction from the suitable articles was conducted using a prebuilt table comprising the first author’s name, the origin of the study, study design, total subjects, and population of the study. For meta-regression purposes, several confounding factors such as age, sex, hypertension, diabetes mellitus, dyslipidemia, smoking status, and follow-up time were also included in the data gathering procedure.

### Risk of bias assessment

The risk of bias assessment for each and every study was performed independently by two authors using the Newcastle–Ottawa Scale (NOS) (11). A study with a total score of seven or above is considered to have a minimal risk of bias. Otherwise, a study with a total score of six or less was found to have a considerable risk of bias and was removed from the study selection process. Quality rating disagreements were resolved by discussion with a third reviewer.

### Data analysis

Software for Statistics and Data Science (STATA) software version 17.0 was utilized to conduct statistical analysis. This meta-analysis’ effect size was expressed as risk ratios (RRs) with 95% confidence intervals (CIs). Any studies that report hazard ratios (HRs) as their effect size were recast into RRs for the purposes of this analysis. The restricted maximum-likelihood method and random-effects models were used to pool the overall effect size despite their heterogeneity. All statistical analyses were two-sided, with statistical significance indicated by a *p*-value <0.05. The *I*^2^ method was used to assess inter-study heterogeneity, and an *I*^2^ statistic >50% or *p*-value <0.10 indicated substantial heterogeneity.

Meta-regression for possible confounders mentioned in the previous subsection was also performed to ensure the true direct effect regarding the aforementioned exposure. Subgroup analyses were also conducted based on the timing and frequency of PVCs to produce an ideal, more robust summary across the relevant studies. The timing of PVCs was divided based on when the PVCs appeared throughout the exercise stress test (either exercise or recovery phase). Furthermore, frequent PVCs were classified as those occurring more than 5 beats per min or accounting for more than 10% of total ventricular depolarizations during the electrocardiographic recordings. We also employed diagnostic test accuracy (DTA) meta-analysis to generate diagnostic values across the statistically significant effect sizes and subgroups, which consisted of sensitivity, specificity, and area under the curve (AUC). Begg’s funnel plot and Egger’s test were used to assessing the likelihood of publication bias in both qualitative and quantitative terms, respectively.

## Results

### Baseline characteristics

**FIGURE 1:**
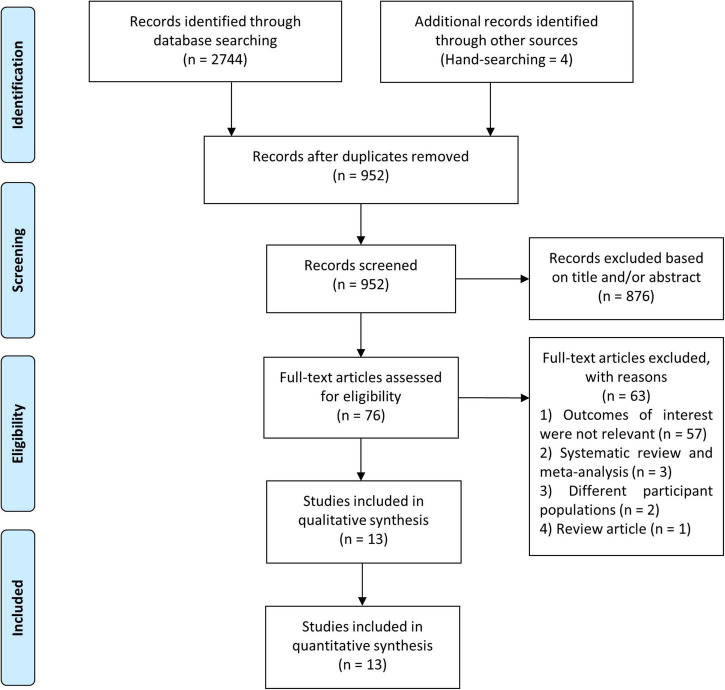
Flow chart of study selection.

We identified 2,748 studies in our initial search, four of which were discovered via the hand-searching procedure, and 952 studies remained after duplicates were removed. Due to the fact that we used the minimum keywords in order to maximize the search results, the number of research dropped dramatically from 952 studies to 76 studies. During this pre-inclusion screening, the majority of the eliminated studies comprised PVCs that did not occur during the exercise stress test, pharmacological trials, diverse study populations, and animal studies. Conclusively, following a screening procedure based on pre-determined inclusion criteria, thirteen studies with a total of 82,161 participants were incorporated in this meta-analysis (Figure 1) (7, 8, 12–22). The mean age of participants in this study was 49.3 years, whereas 69.4% of the participants were male. The prevalence of EI-PVCs in our study was 19.6%. Most of our included articles were conducted in the United States, with a mean duration of follow-up of 11.2 years. All studies provided reasonably clear descriptions of participants, operational definitions, and exposures. Detailed information on the included studies was provided in Table 1.

**TABLE 1:** Characteristics of the included studies.

No.	Author (year)	Country	Study design	Totial partici- pants (*n*)	Study popula-tion	Age (Mean ± SD) (years)	Male (%)	Hyper- tensio*n* (%)	Diabetes mellitus (%)	Dyslipi-daemia (%)	Smoking (%)	Out- come(s)	PVCs preva- lence (%)	Frequent PVCs criteria	Timing of PVCs	Follow-up length (years)	NOS
1	Abdalla et al. (12)	United States	Prospective cohort	15,481	Asymptomatic healthy male	49 ± 6	100	N/A	N/A	N/A	34	ACM, CVM	4.4	≥2 PVCs in 2 min	Exercise phase	7.5	9
2	Aktas et al. (13)	United States	Prospective cohort	3,554	Asymptomatic individuals	57 ± 4.3	81	6	2	33	3	ACM	3	≥7 PVCs in 1 min	Recovery phase	8	9
3	Dewey et al. (14)	United States	Prospective cohort	1,847	Individuals without heart failure who were referred for an exercise test	56 ± 4.2	95.8	15.7	13.3	3	29.5	ACM, CVM	67.6	>10% of total ventricular depolarizations in 30s	Exercise and recovery phase	5.4	9
4	Dhoble et al. (15)	United States	Retrospective cohort	6,546	Individuals without known cardiovascular disease	49.3 ± 2.3	58	24	9	6	15	ACM	31	>5 PVCs in 1 min	Exercise phase	8.1	9
5	Frolkis et al. (16)	United States	Prospective cohort	29,244	Symptom-limited exercise test individuals	56 ± 11	70	45	9	19	16	ACM	5.6	≥7 PVCs in 1 min	Exercise and recovery phase	5.3	9
6	Fuchs et al. (17)	Israel	Retrospective cohort	284	Asymptomatic individuals	25 ± 6	78	N/A	N/A	N/A	N/A	ACM	47	N/A	Exercise phase	5.8	9
7	Jouven et al. (18)	United States	Prospective cohort	6,101	Asymptomatic male without known cardiovascular disease	47.6 ± 1.7	100	15	4.3	4.5	11.7	ACM, CVM	5.1	>10% of total ventricular depolarizations throughout the test	Exercise and recovery phase	23	9
8	Lindow et al. (19)	Sweden	Retrospective cohort	3,106	Symptom-limited exercise test individuals	59 ± 16	54.5	16.3	10	N/A	N/A	CVM	42.7	>10 PVCs in 1 min or >10% of total ventricular depolarizations throughout the test	Recovery phase	7.9	8
9	Myers et al. (20)	United States	Prospective cohort	2,534	Male individuals without known cardiovascular disease	55.5 ± 11.8	100	19.3	N/A	N/A	N/A	ACM	N/A	>10% of total ventricular depolarizations throughout the test	Exercise phase	6.2	9
10	Mora et al. (21)	United States	Prospective cohort	2,994	Asymptomatic female without known cardiovascular disease	45.5 ± 10.5	0	17	2	13	34	ACM, CVM	7.3	>10% of total ventricular depolarizations throughout the test	Exercise phase	20.3	9
11	Morshedi-Meibodi et al. (7)	United States	Prospective cohort	2,885	Asymptomatic individuals without known cardiovascular disease	43 ± 10	55	23	3	3.5	35	ACM	13.6	>10% of total ventricular depolarizations throughout the test	Exercise phase	15	9
12	Marine et al. (8)	United States	Prospective cohort	2,099	Symptom-limited exercise test individuals without known cardiovascular disease	52 ± 12	52.2	33	3.8	12.6	3.8	ACM	3.8	≥2 consecutive PVCs or >10% of total ventricular depolarizations throughout the test	Exercise phase	13.5	9
13	Refaat et al. (22)	United States	Prospective cohort	5,486	Asymptomatic individuals	45.4 ± 10.8	58	43.3	3.2	33	64.6	ACM, CVM	4.3	>10 PVCs in 1 min	Exercise and recovery phase	20.2	9

ACM, all-cause mortality; CAD, coronary artery disease; CVM, cardiovascular mortality; HF, heart failure; MI, myocardial infarction; N/A, not available; NOS, Newcastle-Ottawa Scale; PVCs, premature ventricular complexes; SD, standard deviation.

### Meta-analysis of exercise-induced premature ventricular complexes and poor prognosis

**FIGURE 2:**
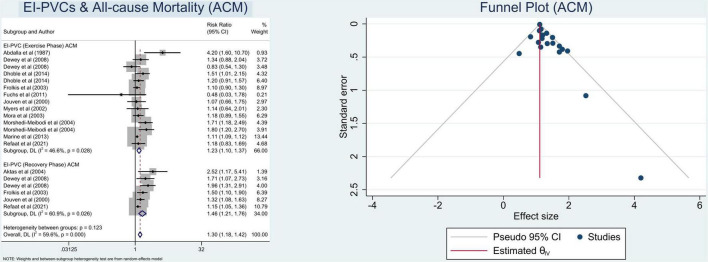
Pooled risk ratios for exercise-induced premature ventricular complexes (EI-PVCs) and all-cause mortality (ACM).

**FIGURE 3:**
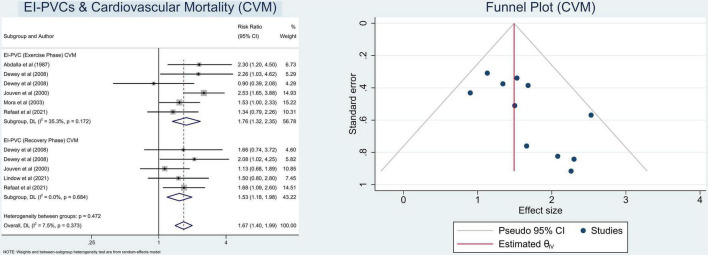
Pooled risk ratios for EI-PVCs and cardiovascular mortality (CVM).

In this meta-analysis, we observed that patients who exhibit EI-PVCs during an exercise stress test have a greater risk of ACM (RR = 1.30 (95% CI = 1.18–1.42); *p* < 0.001; *I*^2^ = 59.6%, *p*-heterogeneity < 0.001) (Figure 2) and CVM (RR = 1.67 (95% CI = 1.40–1.99); *p* < 0.001; *I*^2^ = 7.5%, *p*-heterogeneity = 0.373) (Figure 3). The statistically significant overall combined risk ratio of ACM remained unchanged throughout subgroup analyses, as evidenced by 14 studies reporting EI-PVCs during exercise phase (RR = 1.23 (95% CI = 1.10–1.37); *p* < 0.001; *I*^2^ = 46.6%, *p*-heterogeneity = 0.028), as well as during recovery phase (RR = 1.46 (95% CI = 1.21–1.76); *p* < 0.001; *I*^2^ = 60.9%, *p*-heterogeneity = 0.026), are related with an elevated risk of ACM. Furthermore, subgroup analyses in the CVM outcome show a substantial increase in the risk of CVM in those who had EI-PVCs during the exercise phase (RR = 1.76 (95% CI = 1.32–2.35); *p* < 0.001; *I*^2^ = 35.3%, *p*-heterogeneity = 0.172) as well as people who had EI-PVCs during the recovery period (RR = 1.53 (95% CI = 1.18–1.98); *p* = 0.001; *I*^2^ = 0.0%, *p*-heterogeneity = 0.684).

Information on the specific subtypes of the PVCs (frequency of PVCs, period of PVCs) that occurred within the overall included studies was accessible. Thus, we also performed subgroup analyses based on the previously mentioned categorization, which revealed that the overall combined RRs of ACM and CVM regarding frequent PVCs were (RR = 1.27 (95% CI = 1.15–1.39); *p* < 0.001; *I*^2^ = 53.1%, *p*-heterogeneity = 0.006), and (RR = 1.70 (95% CI = 1.42–2.03); *p* < 0.001; *I*^2^ = 3.8%, *p*-heterogeneity = 0.403), respectively, whereas the overall RRs of ACM and CVM in relation to infrequent PVCs were (RR = 1.37 (95% CI = 0.96–1.95); *p* = 0.080; *I*^2^ = 72.0%, *p*-heterogeneity = 0.013), and (RR = 1.41 (95% CI = 0.62–3.20); *p* = 0.411; *I*^2^ = 55.1%, *p*-heterogeneity = 0.135), respectively, which did not reach statistical significance. Table 2 presents the overall findings as well as the results of other various subsets of subgroup analyses.

**TABLE 2:** Results from the subgroup analysis evaluating the mortality risk of exercise-induced premature ventricular complexes (EI-PVCs).

Subgroup(s)	Risk ratio (95% CI)	Heterogeneity	Number of cohorts
**All-cause mortality (ACM)**			
Overall	1.30 (1.18–1.42) (*P* < 0.001)	*I*^2^ = 59.6% (*P* < 0.001)	20
**Period of PVCs**			
Exercise phase	1.23 (1.10–1.37) (*P* < 0.001)	*I*^2^ = 46.6% (*P* = 0.028)	14
Recovery phase	1.46 (1.21–1.76) (*P* < 0.001)	*I*^2^ = 60.9% (*P* = 0.026)	6
**Recurrence of PVCs**			
Frequent	1.27 (1.15–1.39) (*P* < 0.001)	*I*^2^ = 53.1% (*P* = 0.006)	16
Infrequent	1.37 (0.96–1.95) (*P* = 0.080)	*I*^2^ = 72.0% (*P* = 0.013)	4
Follow-up duration			
> = Median (> = 7.5 years)	1.28 (1.15–1.43) (*P* < 0.001)	*I*^2^ = 57.9% (*P* = 0.005)	13
< Median (<7.5 years)	1.32 (1.06–1.65) (*P* = 0.015)	*I*^2^ = 58.3% (*P* = 0.026)	7
**Cardiovascular mortality (CVM)**			
Overall	1.67 (1.40–1.99) (*P* < 0.001)	*I*^2^ = 7.5% (*P* = 0.373)	11
Period of PVCs			
Exercise phase	1.76 (1.32–2.35) (*P* < 0.001)	*I*^2^ = 35.3% (*P* = 0.172)	6
Recovery phase	1.53 (1.18–1.98) (*P* = 0.001)	*I*^2^ = 0.0% (*P* = 0.684)	5
Recurrence of PVCs			
Frequent	1.70 (1.42–2.03) (*P* < 0.001)	*I*^2^ = 3.8% (*P* = 0.403)	9
Infrequent	1.41 (0.62–3.20) (*P* = 0.411)	*I*^2^ = 55.1% (*P* = 0.135)	2
**Follow-up duration**			
> = Median (> = 7.5 years)	1.66 (1.34–2.06) (*P* < 0.001)	*I*^2^ = 22.3% (*P* = 0.259)	7
< Median (<7.5 years)	1.69 (1.14–2.50) (*P* = 0.009)	*I*^2^ = 2.6% (*P* = 0.379)	4

CI, confidence interval; PVCs, premature ventricular complexes.

### Diagnostic values of exercise-induced premature ventricular complexes

**FIGURE 4:**
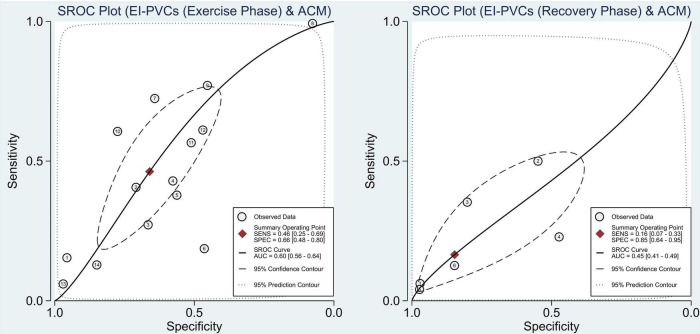
Comparison of SROC curve between two phases regarding ACM. SROC, summary receiver operating characteristics; EI-PVCs, exercise induced premature ventricular complexes; ACM, all-cause mortality; SENS, sensitivity; SPEC, specificity; AUC, area under the curve.

**FIGURE 5:**
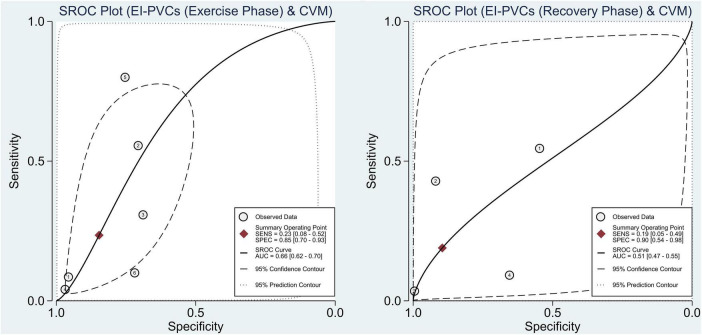
Comparison of SROC curve between two phases regarding CVM. SROC, summary receiver operating characteristics; EI-PVCs, exercise induced premature ventricular complexes; CVM, cardiovascular mortality; SENS, sensitivity; SPEC, specificity; AUC, area under the curve.

With reference to our previous findings that both exercise and recovery phase EI-PVCs were significantly associated with a higher risk of ACM and CVM, summary receiver operating characteristics (SROC) were drawn, and AUC was calculated to determine the diagnostic value of each phase in the exercise stress test. EI-PVCs in the exercise phase had a sensitivity of 46% (25–69%), specificity of 66% (48–80%), and AUC of 60% (56–64%) for ACM outcome. Moreover, it possesses a sensitivity of 16% (7–33%), specificity of 85% (64–95%), and AUC of 45% (41–49%) during the recovery phase. Besides, pertaining to CVM, it was revealed that EI-PVCs in the exercise phase have a sensitivity of 23% (8–52%), specificity of 85% (70–93%), and AUC of 66% (62–70%). Statistical analysis apropos to the recovery phase in CVM demonstrated a sensitivity of 19% (5–49%), specificity of 90% (54–98%), and AUC of 51% (47–55%). Figures 4, 5 illustrate comparisons of the SROC curve, and Table 3 shows the parameters of diagnostic accuracy of each phase for every outcome.

**TABLE 3:** Results of diagnostic value meta-analysis.

Categories	Number of cohorts	Sensitivity (95% CI)	Specificity (95% CI)	AUC (95% CI)
**All-cause mortality (ACM)**				
Overall	20	0.36 (0.20–0.54)	0.73 (0.58–0.84)	0.58 (0.54–0.63)
Period of PVCs				
Exercise phase	14	0.46 (0.25–0.69)	0.66 (0.48–0.80)	0.60 (0.56–0.64)
Recovery phase	6	0.16 (0.07–0.33)	0.85 (0.64–0.95)	0.45 (0.41–0.49)
**Cardiovascular mortality (CVM)**				
Overall	10	0.22 (0.10–0.42)	0.83 (0.70–0.91)	0.61 (0.56–0.65)
Period of PVCs				
Exercise phase	6	0.23 (0.08–0.52)	0.85 (0.70–0.93)	0.66 (0.62–0.70)
Recovery phase	4	0.19 (0.05–0.49)	0.90 (0.54–0.98)	0.51 (0.47–0.55)

CI, confidence interval; PVCs, premature ventricular complexes.

### Meta-regression analysis

An additional meta-regression analysis was carried out to discover if there was a link between prospective covariates and outcomes of interest in this study. The findings revealed that EI-PVCs occurrence and mortality risk were not affected by age, sex, hypertension, diabetes mellitus, dyslipidemia, smoking status, and follow-up time (*p* > 0.05).

### Heterogeneity and risk of bias assessment

The heterogeneity statistic *I*^2^ in this meta-analysis indicates low-to-moderate heterogeneity. There was considerable heterogeneity among the included studies for the ACM subset analysis. Fortunately, after performing a subgroup analysis, the heterogeneity was greatly reduced. With an average NOS score of 8.9 ± 0.3, the Newcastle–Ottawa Scale suggests a minimal likelihood of bias. Eventually, Begg’s funnel plot analysis revealed qualitatively symmetrical funnel plots and there was no indication of small-study effects throughout Egger’s test with regards to the association between EI-PVCs and mortality risk (*p* > 0.05) (Figures 2, 3).

## Discussion

The major findings of this meta-analysis are as follows. Firstly, EI-PVCs increased the risk of ACM and CVM substantially. Secondly, only frequent PVCs are significantly correlated with an elevated risk of ACM and CVM. Thirdly, the specificity of PVCs induced during the recovery phase was higher compared to PVCs that occurred during the exercise phase. Fourthly, EI-PVCs were more significantly associated with ACM and CVM in the short follow-up period (<7.5 years).

Several included cohort studies revealed that patients with EI-PVCs were male, older, smokers, and more likely to have several comorbidities such as hypertension, diabetes mellitus, and dyslipidemia (7, 8, 13, 16, 18, 22, 23). Theoretically, male sex, older age, smoking, and these comorbidities can also contribute to an increase in the risk of mortality in such a population (24–28). Our meta-regression study revealed that the association of EI-PVCs and increased risk of ACM and CVM was not affected by age, sex, hypertension, diabetes mellitus, dyslipidemia, and smoking. Additionally, all included studies performed an adjusted analysis for these covariates. Thus, currently, we can safely infer that EI-PVCs are solely associated with ACM and CVM.

Various hypotheses have been postulated to explain how PVCs induced by exercise testing in an asymptomatic population might enhance the risk of mortality. For starters, the PVC itself can act as the source of ventricular tachyarrhythmia, which increases the risk of sudden cardiac death (SCD) (12). Second, untreated frequent PVCs in the asymptomatic general population can also induce cardiomyopathy, resulting in left ventricular dysfunction and raising the risk of death (29). Our analysis consistently found that, in contrast to infrequent PVCs, frequent PVCs were associated with a considerably elevated risk of ACM and CVM.

As the adage goes “structural heart disease may be silent and go undetected,” it might be one of the foremost inimical risk factors for mortality, given the majority of the included studies did not complete the imaging test at the beginning of the studies (8, 12–15, 18, 20–22). According to two cohort studies conducted by Frolkis et al. (16) and Lindow et al. (19), PVCs were only related to an elevated risk of death when structural heart dysfunctions were present, implying that baseline echocardiography testing may be necessary. However, another large cohort study carried out by Morshedi-Meibodi et al. (7) found that echocardiographic abnormalities were not attributed to an increased risk of mortality in their adjusted-model analysis. As a consequence of these variable results, it is unclear whether structural heart disease raises the risk of mortality in individuals displaying frequent EI-PVCs, suggesting that further studies are required for reassurance. Furthermore, a large cohort study revealed that a group of apparently healthy subjects with no risk factors for CAD had a 24% probability of aberrant stress pattern on an ECG, which is denoted as one of the most critical factors in prognosticating mortality (30). This emphasizes the fact that asymptomatic ischemia is frequent in various clinical populations, which might be a possible source of bias in this investigation. Fortunately, the majority of the studies we considered had already accounted for various potential covariates, including ischemic ECG changes, thereby eliminating this confounding bias (7, 8, 13–16, 18–22).

In the asymptomatic population, PVCs are denoted as a consequence of increasing automaticity in dormant ventricular pacemakers or can be generated by cardiotoxic drugs (e.g., digoxin) or electrolytes disturbances (e.g., hypokalemia and hypomagnesemia) (31). Meanwhile the sympathetic and parasympathetic nervous systems are involved in the underlying mechanism of PVCs created during exercise and recovery phases (32). Exercise-induced PVCs are generated by a surge of catecholamine levels due to sympathetic stimulation during exercise (33). Hayashi et al. study comprising seven patients without structural heart disease discovered that sympathetic predominance can initiate PVCs, resulting in ventricular tachycardia (VT) (34).

Whereas parasympathetic dysfunction through vagal activity impairment can occur in certain individuals during the recovery phase, creating a pro-arrhythmic substrate that leads to PVCs (35). A meta-analysis conducted by Qiu et al. demonstrated that regardless of the presence of PVCs, parasympathetic dysfunction as manifested by attenuation of heart rate recovery was correlated with an increased risk of ACM (35). In a study of 12 individuals without CAD, Osaka et al. discovered that decreased parasympathetic activity can cause non-sustained ventricular tachycardia (36). Moreover, Fei et al. study evaluating 23 patients without heart disease also revealed that idiopathic ventricular tachycardia occurred mostly by attenuation of parasympathetic activity rather than sympathetic activation (37). This result is aligned with our findings, which revealed that PVCs that occurred in the recovery phase disclosed better specificity compared to the exercise phase in predicting the likelihood of ACM and CVM. In contrast to sympathetic stimulation, it appears that parasympathetic dysfunction plays a more prominent role in the development of idiopathic ventricular tachyarrhythmia produced by PVCs and leads to greater mortality risk. Further to that, autonomic nervous system dysfunction can lead to insulin resistance, oxidative stress, and inflammation, all of which contribute to the pathogenesis of cardiovascular diseases, metabolic dysregulation, and other systemic disorders, ultimately increasing the risk of ACM and CVM (38–40).

This meta-analysis indicates that EI-PVCs have poor sensitivity but good specificity in accordance with the diagnostic test accuracy meta-analysis. Thus, it is better utilized to rule in rather than rule out the probability of the aforementioned outcomes of interest. However, it should be noted that the mean age in the majority of the included studies only varied from 43 to 59 years. Thereby, the association between EI-PVCs and mortality in other age groups remained equivocal. We strongly suggest that exercise ECG, as a convenient and widely available test, be performed as a routine auxiliary test in the middle-aged asymptomatic population in order to evaluate the frequency of PVCs and stratify the risk of mortality.

This study offers various advantages over previous meta-analyses. Firstly, the majority of the included studies (10 of 13 studies) have prospective designs, that diminish the risk of recall and selection bias. Secondly, in comparison to the Kim et al. study, this study contained a sufficient number of studies to perform the analysis between PVCs during the recovery phase and interest outcomes, thus the results were able to answer the cause of the moderately high inter-study heterogeneity in the previous meta-analysis. Lastly, the fact that all of our included studies employed multivariable-adjusted models, specifically ischemic ECG changes, further reinforces the value of this study’s pooled risk estimates, demonstrating that EI-PVCs may be used as a prognosticator in predicting mortality.

However, this study still had several major limitations. First, undetected structural heart disease at the baseline could still partly explain the link between EI-PVCs and mortality. Second, low-to-moderate heterogeneity in the ACM analysis may be attributed to variable criteria to classify PVCs across the included studies. Third, due to the majority of included studies enrolling middle-aged participants, the correlation between EI-PVCs and mortality in the younger and older populations remained unclear. Therefore, future prospective cohort studies consisting of age groups other than this range with fixed frequent PVCs criteria and without structural cardiac disease screened by imaging modalities are needed to address the aforementioned issues.

## Conclusion

This study demonstrated that EI-PVCs in both phases (exercise and recovery) substantially raised the risk of ACM and CVM. Nonetheless, only frequent PVCs were shown to be correlated with ACM and CVM. Furthermore, the specificity of PVCs elicited during the recovery phase in predicting the interest outcomes is superior compared to the PVCs in the exercise phase. Hence, we propose that the exercise ECG be employed as an adjunct test in the middle-aged asymptomatic populations to measure the frequency of PVCs and stratify the risk of mortality.

## Data availability statement

The raw data supporting the conclusions of this article will be made available by the authors, without undue reservation.

## Author contributions

MIQ and ICSP conceived and designed the study. ICSP, WK, and RP performed study selection, data extraction, and interpreted the data. MIQ, ICSP, WK, and RP performed extensive search of relevant topics. ICSP, WK, and RP performed the statistical analysis. CA, GK, MP, HG, MRA, ASK, and YHK performed review and extensive editing of the manuscript. All authors contributed significantly to the writing of the manuscript and approved the final manuscript.
